# Exosomes‐transferred LINC00668 Contributes to Thrombosis by Promoting NETs Formation in Inflammatory Bowel Disease

**DOI:** 10.1002/advs.202300560

**Published:** 2023-08-17

**Authors:** Long Zhang, Bin Zheng, Yang Bai, Jing Zhou, Xin‐hua Zhang, Yu‐qin Yang, Jing Yu, Hong‐ye Zhao, Dong Ma, Han Wu, Jin‐kun Wen

**Affiliations:** ^1^ Department of Biochemistry and Molecular Biology The Key Laboratory of Neural and Vascular Biology Ministry of Education of China Hebei Medical University Shijiazhuang 050017 China; ^2^ Department of Endocrine The Second Hospital of Hebei Medical University Shijiazhuang 050017 China; ^3^ Institution of Chinese Integrative Medicine Hebei Medical University Shijiazhuang 050017 China; ^4^ Department of Respiratory The Second Hospital of Hebei Medical University Shijiazhuang 050017 China

**Keywords:** berberines, exosomes, inflammatory bowel diseases, LINC00668, neutrophil elastase, neutrophil extracellular traps, thrombus

## Abstract

Epidemiological studies show an association between inflammatory bowel disease (IBD) and increased risk of thrombosis. However, how IBD influences thrombosis remains unknown. The current study shows that formation of neutrophil extracellular traps (NETs) significantly increased in the dextran sulfate sodium (DSS)‐induced IBD mice, which in turn, contributes to thrombus formation in a NETs‐dependent fashion. Furthermore, the exosomes isolated from the plasma of the IBD mice induce arterial and venous thrombosis in vivo. Importantly, proinflammatory factors‐exposed intestinal epithelial cells (inflamed IECs) promote neutrophils to release NETs through their secreted exosomes. RNA sequencing revealed that LINC00668 is highly enriched in the inflamed IECs‐derived exosomes. Mechanistically, LINC00668 facilitates the translocation of neutrophil elastase (NE) from the cytoplasmic granules to the nucleus via its interaction with NE in a sequence‐specific manner, thereby inducing NETs release and thrombus formation. Importantly, berberine (BBR) suppresses the nuclear translocation of NE and subsequent NETs formation by inhibiting the interaction of LINC00668 with NE, thus exerting its antithrombotic effects. This study provides a novel pathobiological mechanism linking IBD and thrombosis by exosome‐mediated NETs formation. Targeting LINC00668 can serve as a novel molecular treatment strategy to treat IBD‐related thrombosis.

## Graphical abstract

Inflamed intestinal epithelial cells secrete exosomes containing LINC00668 into the bloodstream, which are uptaken by neutrophils. In the neutrophils, LINC00668 binds to NE released from the azurophilic granules into the cytosol and carries NE to the nucleus from the cytoplasm, where NE cleaves histones and subsequently causes chromatin decondensation, thereby promoting the release of NETs and IBD‐related thrombosis.

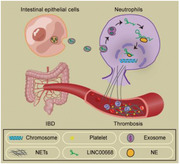

## 1 Introduction

Inflammatory bowel disease (IBD) is a recurrent gastrointestinal disorder classified as Crohn's disease (CD) and ulcerative colitis (UC). IBD can occur at any age, and its prevalence remains high and has gradually spread from developed to developing countries.^[^ ^1^ ^]^ Growing evidence has suggested that patients with IBD are predisposed to thrombosis,^[^ ^2^ ^]^ with ≈3‐ to 4‐fold increase in the risk of venous thrombosis compared with the general population.^[^ ^3^ ^]^ Thrombus formation disrupts normal blood flow in arteries or veins, resulting in several pathologies including myocardial infarction, ischemic stroke, pulmonary embolism, deep vein thrombosis,^[^ ^4^ ^]^ and coronavirus disease 2019‐associated microthrombosis.^[^ ^5^ ^]^ With one in four people dying worldwide from thrombotic conditions, thrombosis is a major contributor to global disease burden.^[^ ^6^ ^]^ Traditionally, the studies on thrombosis are mainly focused on platelets and plasma proteins, and the thrombus is considered to be formed by the interaction of platelets, fibrin, and red blood cells.^[^ ^7^ ^]^ In the last decade, the relationship between neutrophils and thrombosis is gradually unraveled. Specifically, the discovery of neutrophil extracellular traps (NETs) as new DNA‐based players in thrombosis has significantly changed our understanding of the pathophysiology of thrombosis.^[^ ^4^, ^8^ ^]^ Despite increasing knowledge of molecular mechanisms underlying NETs formation, little is known about how IBD is associated with increased NETs formation and thrombosis. Understanding the mechanism of NETs formation is of great significance for the treatment of IBD‐associated thrombosis.

Neutrophils, as the first‐line defense against invading pathogens or harmful agents, are involved in the initiation and propagation of the inflammatory response.^[^ ^9^ ^]^ Under normal physiological conditions, neutrophil elastase (NE) and myeloperoxidase (MPO), which degrade bacterial virulence factors,^[^ ^8^ ^]^ are stored in the azurophilic granules of neutrophils. When neutrophils are activated, NE escapes the granules and enters the nucleus, where it cleaves histones and facilitates chromatin de‐condensation. Then, MPO binds to chromatin to promote further de‐condensation, leading to cell rupture and NETs release.^[^ ^10^ ^]^ NETs, as a double‐edged sword,^[^ ^11^ ^]^ not only function as a physical barrier that prevents the spread of pathogens and kills microbes,^[^ ^8^ ^]^ but can induce thrombus formation by providing a scaffold for adhesion of platelets, red blood cells, and platelet adhesion molecules such as fibrinogen, von Willebrand factor (VWF), and fibronectin.^[^ ^12^ ^]^ Simultaneously, the multiple components themselves of this scaffold, including histones, DNA, NE, cathepsin G, etc, can also trigger platelet activation and blood coagulation through activating intrinsic and extrinsic coagulation pathways.^[^ ^4^, ^13^ ^]^ Indeed, it has been reported that plasma levels of NETs were significantly higher in patients with active IBD than in healthy volunteers and patients with inactive IBD.^[^ ^14^ ^]^ Moreover, the stimulation of neutrophils isolated from healthy individuals with the serum of patients with active IBD induced significant NET release.^[^ ^15^ ^]^

Although the exact mechanisms of NETs formation are still poorly understood, some key steps have been identified, including reactive oxygen species (ROS) production mediated by nicotinamide adenine dinucleotide phosphate (NADPH) oxidase,^[^ ^16^ ^]^ histone guanylation induced by peptidyl deiminase 4 (PAD4),^[^ ^17^ ^]^ and chromatin deprotonation regulated by various proteases such as NE and MPO.^[^ ^18^ ^]^ Despite intensive investigation, much remains unknown about mechanisms linking inflammation of the intestinal epithelium in IBD to NETs formation that occurs in IBD. Exosomes or extracellular vesicles play a crucial role in intercellular communication by acting as carriers of functional contents such as proteins, RNAs, lipids, and other biomolecules. Several studies have reported that mRNAs and proteins in extracellular vesicles isolated from plasma,^[^ ^19^ ^]^ saliva,^[^ ^20^ ^]^ and intestinal lumen^[^ ^21^ ^]^ of IBD patients have pro‐inflammatory potential to cause an inflammatory response in colonic epithelial cells and macrophages. Similar findings were also obtained in IBD mouse model, where serum‐derived exosomes from acute colitis mice versus control mice contained a variety of differentially expressed proteins, most of which are associated with complement, coagulation cascade, and macrophage activation.^[^ ^22^ ^]^ However, it is still poorly understood how these exosomes and their active biomolecules may contribute to NETs formation in the context of IBD.

In the present study, we hypothesized that exosomes secreted by inflamed intestinal epithelial cells (IEC) may promote thrombus formation by inducing the release of NETs. We identified important biomolecules responsible for exosome‐induced NETs formation and explored the underlying mechanism of how the IEC‐derived exosomes contribute to NETs formation and thrombosis.

## 2 Results

### 2.1 Increased Thrombosis is Associated with NETs Formation in Mice with DSS‐Induced Intestinal Inflammation

Epidemiological studies show that patients with IBD have a higher incidence of thrombosis than the general population.^[^ ^2^, ^23^ ^]^ To investigate whether this increased risk of blood clots in human IBD is recapitulated in rodent IBD models, we used 8‐week‐old female BALB/c mice to establish IBD models by drinking water containing 3% dextran sodium sulfate (DSS) for 7 days, while mice in the control group drank ordinary sterilized water. Body weight, stool consistency, and the gross blood present in feces and near the anus were monitored every day. Significant weight loss occurred in DSS‐treated mice from day 4, accompanied by rectal bleeding and diarrhea (Figure S1a,b, Supporting Information). Histological analyses of distal colon sections showed that DSS‐treated mice exhibited severe inflammation relative to control mice, as evidenced by inflammatory cell infiltration, crypt loss, and increased ulceration (Figure S1c,d, Supporting Information). The colon length of DSS‐treated mice was significantly shorter than that of mice in the control group (Figure S1e,f, Supporting Information). These observations suggest that the IBD model was successfully established in the BALB/c female mice via 3% DSS in drinking water.

**Figure 1:**
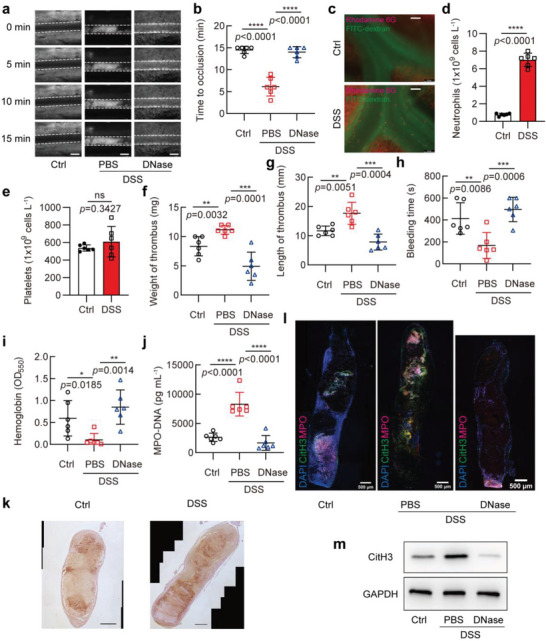
Increased thrombosis is associated with NETs formation in mice with DSS‐induced colitis. a–c) Mesenteric arterioles ≈100 µm in diameter were injured with FeCl_3_ and white blood cells and platelets were labeled with Rhodamine 6G to detect thrombus formation by intravital microscopy. a) Representative images of thrombus formation in Ctrl or 3% DSS (PBS)‐treated mice administered with or without DNase I (DNase) via the tail vein. Scale bars represent 100 µm. b) Vascular occlusion time in three groups of mice treated as in (a), with 6 mice in each group. c) Representative image of mesenteric vein with white blood cells and platelets labeled by Rhodamine 6G (red) and blood flow labeled by FITC‐dextran (green). Scale bars represent 50 µm. d) Neutrophil and e) platelet count in plasma of Ctrl and DSS‐treated mice after 7‐day treatment, with 6 mice in each group. EDTA‐anticoagulated whole blood was analyzed by a five‐category blood cell analyzer (Mindray, BC‐5000vet). f,g) Mice treated as in (a) were subjected to surgery for inferior vena cava stenosis, f) weight and g) length of thrombus harvested from 3 groups of mice (6 mice in each group) were measured. h) Tail bleeding time in mice treated as in (a), with 6 mice in each group. i) Blood loss was measured by the absorbance of hemoglobin, which was released from ruptured erythrocytes, at a wavelength of 550 nm using a microplate reader, with 6 mice in each group, and each mouse performed three replicates. j) MPO‐DNA ELISA was used to assess NETs in plasma of three groups of mice treated as in (a), with 6 mice in each group. k) Representative immunohistochemical staining images of Ly6G, scale bars, 500 µm. l) Citrullinated histone H3 (CitH3, green) and MPO (magenta) staining in the thrombus of inferior vena cava in three groups of mice treated as in (a) (*n* = 3 mice per group). Scale bars, 500 µm. m) Western blot analysis of CitH3 expression in the whole thrombus obtained two days after surgery for inferior vena cava stenosis. Data are represented as mean ± SEM, ns means no significance, *p<0.05, **p<0.01, ***p<0.001, ****p<0.0001, p‐value was determined by unpaired two‐tailed Student's t‐test.

Next, we used the superior mesenteric artery with a diameter of ≈100 µm to investigate whether mice with IBD are more prone to thrombosis. To do this, the leukocytes and platelets in the peripheral blood of mice were labeled by injecting Rhodamine 6G into the tail vein, thrombus formation in FeCl_3_‐injured mesenteric arterioles was monitored by intravital microscopy, and the time to complete blood flow occlusion was recorded. We found that mice treated with DSS had shorter vascular occlusion times than those in control mice (**Figure** **1**a,b). Notably, there were significantly more Rhodamine 6G‐positive cells in the superior mesenteric artery in the DSS‐treated mice than in the control mice (Figure 1c). To identify cell type of rhodamine 6G‐positive cells, we performed peripheral blood cell analysis (Table S1, Supporting Information). As shown in Figure 1d,e, the number of neutrophils increased significantly in the DSS‐treated mice, while platelet counts were not statistically different between the two groups. Next, we observed the thrombus formation through the inferior vena cava stenosis model, a widely used model to induce deep vein thrombosis in mice, and took out the thrombus for measurement 48 h after the operation. The results showed that both weight and length of the thrombus were significantly increased in the DSS‐treated mice compared with controls (Figure 1f,g). Moreover, tail bleeding time and hemoglobin level in the DSS‐treated mice were reduced (Figure 1h,i). These results clearly suggest that the mice with DSS‐induced colitis are more prone to thrombosis than control mice.

Neutrophils, the most abundant type of leukocytes in blood, play a crucial role in promoting thrombosis via NETs formation,^[^ ^24^ ^]^ and there was a higher level of plasma NETs in patients with active IBD than in healthy participants.^[^ ^14^, ^25^ ^]^ Therefore, we evaluated whether the level of plasma NETs was elevated in the DSS‐treated mice and found that MPO‐DNA complexes, as markers of NETs, were significantly increased in the DSS‐treated group when compared to control group (Figure 1j). To further clarify whether the increased NETs formation in peripheral blood is responsible for thrombosis, we detected the content of neutrophils and NETs in venous thrombus induced by inferior vena cava stenosis. The results showed that compared with the control group, the number of Ly6G‐positive cells (Figure 1k) and the content of NETs (Figure 1l), as shown by coincident staining for citrullinated histone H3 (CitH3) and MPO, markedly increased in the venous thrombus of the mice with the DSS‐induced colitis. Further, we determined the content of NETs in the whole blood thrombus by detecting the NETs marker protein CitH3 after the thrombus was homogenized. Western blot analysis showed a dramatically increased amount of CitH3 that was robust two days after surgery in the DSS‐treated group (Figure 1m).

To further explore the involvement of NETs released by neutrophils in IBD‐induced thrombosis, we used DNase I to degrade DNA in NETs by mouse tail vein injection. The results showed that application of DNase I resulted in a marked reduction of the content of NETs in the thrombus (Figure 1j,l,m). Correspondingly, the above‐mentioned observations in the DSS‐treated group, including vascular occlusion time (Figure 1b), weight and length of the thrombus (Figure 1f,g), bleeding time, and hemoglobin levels (Figure 1h,i) were greatly restored by treating with DNase I. Based on this finding that susceptibility of DSS‐treated mice to thrombosis was easily suppressed by DNase I, we concluded that neutrophils promote thrombus formation in a NETs‐dependent fashion.

### 2.2 Exosomes Secreted into Plasma from DSS‐Induced IBD Mice Play an Important Role in the IBD‐Related Thrombosis

**Figure 2:**
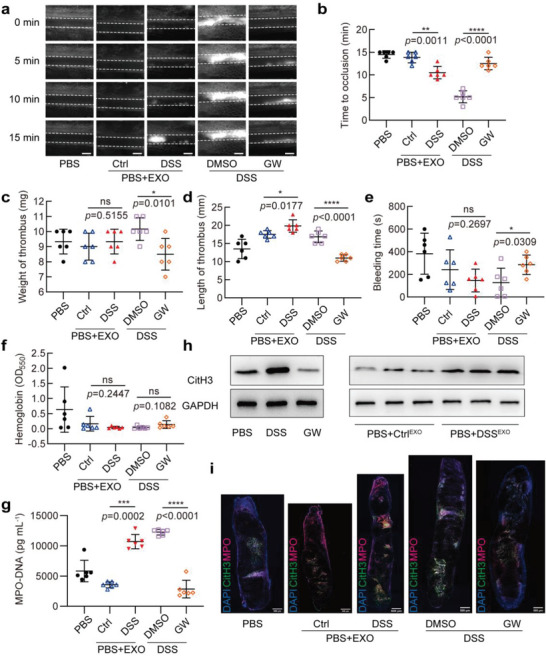
Exosomes secreted into plasma from DSS‐induced IBD mice promote thrombosis. a,b) Mesenteric arterioles ≈100 µm in diameter were injured with FeCl_3_ and white blood cells and platelets were labeled with Rhodamine 6G to detect thrombus formation by intravital microscopy. a) Representative images of thrombus formation in five different groups of mice treated as follows: mice were given drinking water freely (Ctrl), treated with PBS (PBS), 3% DSS (DSS), or 3% DSS combined with vehicle (DMSO) or GW4869 (GW) via intraperitoneal injection. Among these five groups, Ctrl and DSS groups were administrated with exosomes isolated from Ctrl or DSS‐treated mice through the tail vein. Scale bars represent 100 µm. b) Vascular occlusion time in 5 groups of mice treated as in (a), with 6 mice in each group. c,d) Mice treated as in (a) were subjected to surgery for inferior vena cava stenosis, after 48 h, c) weight and d) length of thrombus harvested from 5 groups of mice (6 mice in each group) were measured. e) Tail bleeding time in 5 groups of mice treated as in (a), with 6 mice in each group. f) Blood loss was measured in 5 groups of mice by the absorbance of hemoglobin at a wavelength of 550 nm using a microplate reader (6 mice in each group). g) MPO‐DNA ELISA was used to assess NETs in plasma of 5 groups of mice treated as in (a), with 6 mice in each group. h) Mice treated with PBS were administrated with exosomes isolated from Ctrl (Ctrl^EXO^) or DSS‐treated (DSS^EXO^) mice through the tail vein, and Western blot analysis of CitH3 expression was performed in the whole thrombus obtained two days after surgery for inferior vena cava stenosis. i) CitH3 (green) and MPO (magenta) staining in the thrombus of inferior vena cava in 5 groups of mice treated as in (a) (*n* = 3 mice per group). Data are represented as mean ± SEM, ns means no significant difference, *p<0.05, **p<0.01, ***p<0.001, ****p<0.0001, p‐value was determined by unpaired two‐tailed Student's t‐test.

The above findings prompted us to study how IBD affects thrombus formation. Previous studies have shown that the active ingredients contained in exosomes from mice with colitis can remotely regulate the functions of fibroblasts, macrophages, and T cells.^[^ ^22^, ^26^ ^]^ Therefore, we hypothesized that exosomes might bridge IECs to neutrophils. We next investigated whether exosomes were involved in DSS‐induced thrombogenesis in mice with colitis. We used GW4869, which is the most widely used inhibitor of exosome generation,^[^ ^27^ ^]^ to inhibit exosome release. The results revealed that exosomes from the DSS‐treated mice significantly shortened vascular occlusion times compared with those from control group. Compared with DMSO group, administration of GW4869 to the DSS‐treated mice significantly prolonged the occlusion time of superior mesenteric artery (**Figure** **2**a,b). Also, both weight and length of thrombi harvested from the inferior vena cava were lower in the GW4869‐treated groups than those of GW4869‐untreated groups (Figure 2c,d). And the tail‐bleeding time was prolonged, however, blood loss was not significantly different between the two groups of mice (Figure 2e,f). Then we detected the content of NETs in the peripheral blood and venous thrombus. As expected, GW4869 treatment diminished the levels of MPO‐DNA complexes in peripheral blood (Figure 2g). Consistently, the content of NETs in blood clots was also reduced, as shown by decreased CitH3 level as well as by reduced coincident staining for CitH3 and MPO (Figure 2h,i). These results indicate that inhibition of the exosome release using GW4869 can delay the thrombus formation in the DSS‐treated mice by reducing the level of NETs in peripheral blood and venous thrombosis.

Additionally, to further clarify the role of exosomes in thrombosis, mouse plasma exosomes were collected using iodixanol density gradient centrifugation (Figure S2a, Supporting Information). The collected exosomes were detected by Western blotting for exosomal marker proteins ALIX, TSG101, and CD9 (Figure S2b, Supporting Information). The size and concentration of exosomes were measured using nanoparticle tracking analysis (Figure S2c, Supporting Information). The results indicated that the marker proteins and size distribution of the exosomes purified from mouse plasma samples were consistent with the known characteristics of exosomes. We treated mice twice a week with exosomes from mouse plasma of the control and DSS‐treated groups by injecting via tail vein. Followed by using FeCl_3_‐induced superior mesenteric artery injury or inferior vena cava ligation, we examined the formation of arterial and venous thrombosis. The results showed that mice receiving the exosomes from DSS‐treated mice were more prone to thrombosis than control mice (Figure 2a‐f), and higher levels of NETs were detected in peripheral blood and venous thrombosis (Figure 2g‐i). Taken together, the exosomes secreted into plasma from DSS‐induced colitis mice can promote the thrombus formation in a NETs‐dependent manner, and the inhibition of exosome secretion can delay the thrombosis to a certain extent.

### 2.3 Inflamed IECs Induce Neutrophils to Generate NETs

**Figure 3:**
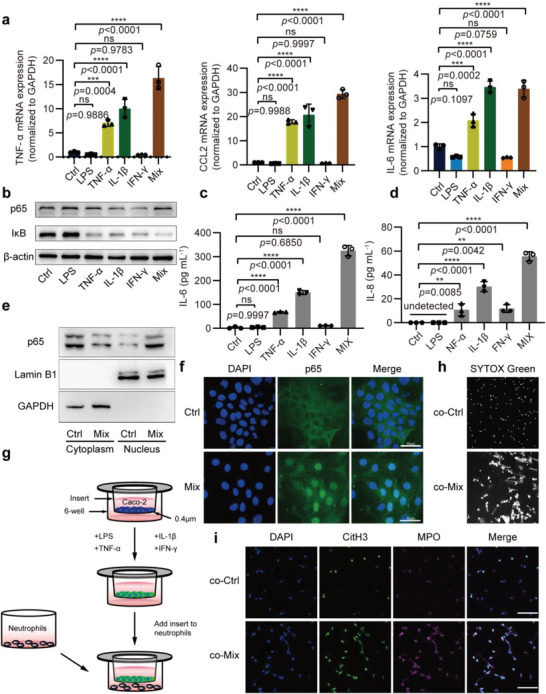
Inflamed IECs induce neutrophils to generate NETs. a) qPCR analysis for TNF‐α, CCL2, and IL‐6 mRNA in Caco‐2 cells treated with PBS (Ctrl), 10 µg mL^−1^ LPS (LPS), 50 µg mL^−1^ TNF‐α (TNF‐α), 25 µg mL^−1^ IL‐1β (IL‐1β), 50 µg mL^−1^ IFN‐γ (IFN‐γ) or a mixture of the above four factors (Mix). b) Western blot analysis of p65 and IκB expression in 6 groups of Caco‐2 cells treated as in (a). c,d) ELISA assay of IL‐6 and IL‐8 in the culture supernatant of 6 groups of Caco‐2 cells treated as in (a). e) Western blotting analysis determined nuclear and cytoplasmic expression of NFκB p65 in Caco‐2 cells treated with PBS (Ctrl) or Mix. f) p65 (green) staining in Caco‐2 cells treated with PBS (Ctrl) or Mix, the nuclei are stained with DAPI (blue). g) Schematic diagram of the Transwell co‐culture system for Caco‐2 cells (upper chamber) and neutrophils (lower chamber). h) Representative image of extracellular DNA stained with SYTOX Green in neutrophils after co‐cultured with Caco‐2 cells treated with Ctrl or Mix. i) CitH3 (green) and MPO (magenta) staining in neutrophils that co‐cultured with Caco‐2 cells treated with Ctrl or Mix, the nuclei are stained with DAPI (blue). Data are represented as mean ± SEM, ns means no significant difference, **p<0.01, ***p<0.001, ****p<0.0001, *p*‐value was determined by one‐way ANOVA with Dunnett's post hoc correction.

Because IBD is known to be a chronic inflammation of the intestinal tract,^[^ ^23a^ ^]^ and inflammation of the intestinal epithelium is an important feature of IBD,^[^ ^28^ ^]^ we sought to determine whether IECs are responsible for NETs formation and IBD‐related thrombosis. Thus, we selected Caco‐2 cells, a human intestinal cell line, to perform in vitro experiments because this cell line can differentiate into polarized monolayers after fusion, similar in structure to normal IECs.^[^ ^29^ ^]^ First, we established an inflammatory model of Caco‐2 cells induced by proinflammatory factors. Caco‐2 cells were treated with LPS (10 µg mL^−1^), TNF‐α (50 ng mL^−1^), IL‐1β (25 ng mL^−1^), IFN‐γ (50 ng mL^−1^), respectively, as well as with a mixture of these four factors to induce inflammation. Quantitative PCR showed that both TNF‐α and IL‐1β could effectively promote the inflammatory response in Caco‐2 cells, as manifested by significantly increased levels of TNF‐α, CCL2, and IL‐6 (**Figure** **3**a). And IκB expression was noticeably decreased, whereas NF‐κB p65 expression was only slightly elevated (Figure 3b). ELISA assay of IL‐6 and IL‐8 from the culture supernatant confirmed that TNF‐α and IL‐1β were strong inducers of Caco‐2 cell inflammation (Figure 3c, d). However, the expression of inflammatory factors induced by LPS and IFN‐γ was not obvious (Figure 3a,c,d). When the above four factors (named as Mix) were combinatively used, the inflammatory response of Caco‐2 cells was further increased (Figure 3a‐d). In order to determine whether the p65 in the Mix‐treated Caco‐2 cells enters the nucleus to activate inflammatory gene transcription, we extracted the cytoplasmic protein and nuclear protein, respectively. Compared with the control group, the nuclear distribution of p65 was significantly increased in the Mix‐treated Caco‐2 cells (Figure 3e). This was also confirmed by immunofluorescence staining, showing that the fluorescent signals of p65 markedly accumulated in the nucleus of Mix‐treated Caco‐2 cells (Figure 3f). The above results indicate that inflammatory model of Caco‐2 cells was successfully induced by a mixture of the four inflammatory factors. We used this mixture to treat Caco‐2 cells in all subsequent experiments.

Next, we conducted co‐culture experiments with Caco‐2 cells and neutrophils, in which the two cells were separated by a membrane of 0.4 µm pore size to prevent direct cell contact but allow the exosomes to pass. In this setup, Caco‐2 cells were placed in the upper chamber of a co‐culture system, and the lower chamber was neutrophils (Figure 3g). Caco‐2 cells on the upper layer were treated with Mix for 24 h and then co‐cultured with neutrophils for 4 h. Subsequently, we treated neutrophils with membrane‐impermeable DNA dye SYTOX green, which binds DNA released from cells when the cellular membranes were damaged, resulting in green fluorescence. Thus, NETs‐producing cells, whose cellular membranes were damaged and that DNA was released, showed extracellular filamentous DNA structures. As a result, Mix‐treated Caco‐2 cells could induce the release of large amounts of filamentous extracellular DNA from neutrophils (Figure 3h). These filamentous structures were further identified as NETs by double fluorescent staining of CitH3 and MPO (Figure 3i). Collectively, these data indicate that inflamed IECs can induce neutrophils to produce NETs.

### 2.4 Inflamed IECs‐derived Exosomes Promote Neutrophils to Release NETs

**Figure 4:**
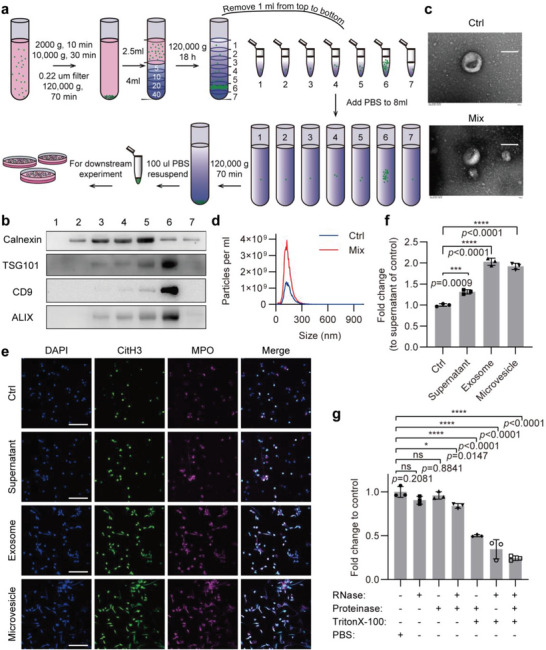
Inflamed IECs‐derived exosomes promote neutrophils to release NETs. a) Schematic of exosome preparation from cell culture supernatant of Ctrl or Mix‐treated Caco‐2 cells. b) Western blot analysis detected the ER‐associated protein calnexin and the exosome markers TSG101, CD9, and ALIX in 7 fractions during exosome isolation. c) Electron microscopic image of exosomes isolated from Ctrl or Mix‐treated Caco‐2 cells. Scale bars represent 100 nm. d) Nanoparticle Tracking Analysis of exosomes isolated from Ctrl or Mix‐treated Caco‐2 cells. e) Immunofluorescence staining for CitH3 (green) and MPO (magenta) was performed on neutrophils treated with supernatant, exosome, or microvesicle from Mix‐treated Caco‐2 cells. Scale bars represent 50 µm. f) Quantification of NETs release by measuring the OD of SYTOX Green at 523 nm, fold change normalized to supernatant of control (Ctrl). g) PBS‐, RNase‐, proteinase‐, RNase + proteinase‐, proteinase + Triton X‐100‐, RNase + Triton X‐100‐, or RNase + proteinase + Triton X‐100‐treated exosomes were added to neutrophils, and then quantification of NETs was detected by measuring the fluorescence of SYTOX Green at Ex485, Em520. Data are represented as mean ± SEM, ns means no significant difference (*p* > 0.05), *p<0.05, ***p<0.001, ****p<0.0001, p‐value was determined by one‐way ANOVA with Dunnett's post hoc correction.

Based on the above experimental results, we hypothesized that Mix‐treated Caco‐2 cells‐derived exosomes might be involved in transferring their bioactive cargos to neutrophils as well as in inducing the formation of NETs. To test this hypothesis, we first used iodixanol density gradient centrifugation to obtain higher yields of pure exosomes from the cell culture supernatants (**Figure** **4**a). The enrichment of exosomes in the 7 fractions from top to bottom was detected by the Western blotting analysis of exosome markers, such as TSG101, CD9, and ALIX. The exosomes were found to be highly enriched in the sixth fraction, and the ER‐associated protein calnexin was essentially free (Figure 4b). Transmission electron microscopy and nanoparticle tracking analysis showed that the exosomes obtained exhibited a round or oval cup shaped‐appearance, and the size of vesicles was distributed between 50 and 200 nm which is considered as ideal size for exosomes (Figure 4c,d). These observations indicate that the isolated exosomes had high purity and can be used for subsequent functional studies. We separated vesicle‐free supernatant, microvesicles, and exosomes from the culture supernatants by differential centrifugation, and then treated neutrophils with them, respectively, and observed the production of NETs. The results showed that compared with the culture supernatants from Mix‐untreated Caco‐2 cells, all three components of the culture supernatants from Mix‐treated Caco‐2 cells stimulated neutrophils to produce NETs, as evidenced by coincident staining for CitH3 and MPO (Figure 4e). Among these three components, the exosomes and microvesicles had a stronger effect on inducing NETs formation than that of the vesicle‐free supernatant. We further used SYTOX Green staining to quantify the amount of the released extracellular DNA and found that neutrophils treated with the Mix‐induced exosomes released more NETs than those treated with the vesicle‐free supernatant or microvesicles (Figure 4f).

The exosomes secreted from the Mix‐treated Caco‐2 cells contained cytokines, growth factors, mRNAs, miRNAs, and other active molecules, and they could also directly induce neutrophils to generate NETs. To determine which active molecules in exosomes are responsible for inducing NETs formation, we performed the exosome degradation assay. Exosomes were treated with RNase, protease, or Triton X‐100 alone or the synergy between them for 4 h, and then were co‐incubated with neutrophils, followed by NETs quantification using SYTOX Green staining. After degradation of the exosomal RNA by RNase and Triton X‐100 synchronously, the ability of exosomes to induce neutrophils to release NETs was greatly reduced, and simultaneous degradation of the exosomal RNA and proteins by RNase and protease further limited their ability to enhance neutrophils to release NETs (Figure 4g). Altogether, these results provide clear evidence demonstrating that exosomes secreted by inflamed Caco‐2 cells directly promote neutrophils to release NETs in an exosomal RNA‐dependent manner.

### 2.5 Identification of LINC00668 as Molecule Responsible for IEC Exosome‐Induced NETs Formation

**Figure 5:**
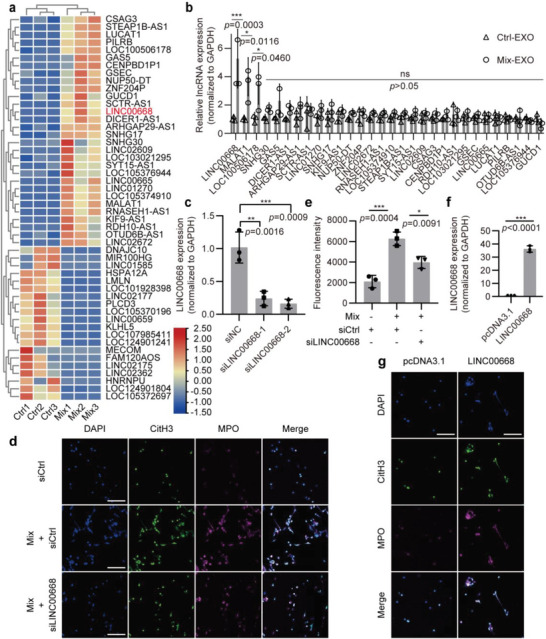
Identification of LINC00668 as molecule responsible for IEC exosome‐induced NETs formation. a) The differentially expressed lncRNAs, which were detected with microarray analysis, in exosomes derived from Ctrl or Mix‐treated Caco‐2 cells were selected and summarized. Heatmap showing lncRNAs with significant change, represented by red (upregulated) and blue (downregulated). b) qRT‐PCR validation of a subset of the significantly up‐regulated lncRNAs. Their relative expression levels were normalized to GAPDH. c) Two siRNAs targeting different regions of LINC00668 were used to knock down LINC00668 in Caco‐2 cells, the knockdown efficiency was detected by qRT‐PCR. d) Caco‐2 cells transfected with siCtrl or siLINC00668 and then treated with Mix were co‐cultured with neutrophils for 4 h, and immunofluorescence staining for CitH3 (green), MPO (magenta), and DAPI (blue) was performed on neutrophils. Scale bars represent 50 µm. e) Quantification of NETs in siCtrl or siLINC00668‐transfected neutrophils was performed by measuring the fluorescence of SYTOX Green at Ex485, Em520. f) The overexpression plasmid of LINC00668 was designed and constructed, and the overexpression efficiency was verified by qRT‐PCR. g) pcDNA3.1 and LINC00668‐overexpressing plasmids were transfected into neutrophils, respectively, and neutrophils were stained with CitH3 (green), MPO (magenta), and DAPI (blue) by immunofluorescence. Scale bars represent 50 µm. Data are represented as mean ± SEM, ns means no significant difference (*p* > 0.05), *p<0.05, **p<0.01, ***p<0.001, p‐value was determined by unpaired two‐tailed Student's t‐test (b, e, f) and one‐way ANOVA with Dunnett's post hoc correction (c).

Emerging evidence suggests that lncRNAs encapsulated by exosomes are the important molecules by which exosomes regulate recipient cell function.^[^ ^30^ ^]^ To identify specific lncRNAs responsible for inflamed IEC exosome‐induced NETs formation, we used high‐throughput sequencing to analyze the composition of lncRNAs in the exosomes secreted by Mix‐treated Caco‐2 cells versus Mix‐untreated cells. A total of 200 lncRNAs were differentially expressed by more than 2‐fold between Mix‐treated Caco‐2 cells and Mix‐untreated cells, including 133 upregulated and 67 downregulated lncRNAs (**Figure** **5**a). Next, the expression levels of 30 significantly upregulated lncRNAs from the RNA‐sequencing data were validated by qRT‐PCR (Figure 5b). Among these significantly upregulated lncRNAs, LINC00668 was ranked on the top based on fold of changes, and this lncRNA has been reported to be implicated in malignant processes of some cancers.^[^ ^31^ ^]^ However, its roles and mechanisms of action in the NETs formation and IBD‐associated thrombosis are unknown. Thus, we focused on LINC00668 in the subsequent experiments. To further investigate the role of the exosomal lINC00668 derived from Caco‐2 cells in the regulation of NETs formation, we first examined whether LINC00668 was also expressed in neutrophils besides intestinal epithelial cells. Intriguingly, LINC00668 was barely detectable in neutrophils regardless of treatment with proinflammatory factor (Figure S3a, Supporting Information). Next, we performed LINC00668 loss‐of‐function and gain‐of‐function studies. The successful knockdown of LINC00668 by two siRNAs in Caco‐2 cells was confirmed by RT‐PCR (Figure 5c). Moreover, siLINC00668 treatment also reduced the content of LINC00668 in the exosomes (Figure S3b, Supporting Information). After LINC00668‐silenced Caco‐2 cells were co‐cultured with neutrophils, NETs formation was detected by immunofluorescence staining for CitH3 and MPO. The results showed that when LINC00668‐silenced Caco‐2 cells were treated with Mix, their ability to promote neutrophils to release NETs was largely lost (Figure 5d). Similar results were obtained by immunofluorescence staining for NETs with SYTOX Green (Figure 5e). Additionally, we constructed LINC00668 expression plasmids, then transfected neutrophils with them, and demonstrated successful overexpression of linc00668 in neutrophils (Figure 5f). We found that overexpression of LINC00668 in neutrophils facilitated NETs release (Figure 5g). Overall, these data clearly suggest that IEC LINC00668 is produced by inflamed Caco‐2 cells and is transferred to neutrophils by exosomes, where it plays an important role in the formation of NETs.

To determine if our experimental findings are relevant to human disease, we collected the samples from 8 patients with IBD and 8 healthy controls and analyzed the expression of LINC00668 and NETs in plasma of these patients and healthy controls. Our preliminary results showed that the level of exosomal‐LINC00668 and the content of NETs were significantly elevated in the plasma of the patients versus healthy controls (Figure S3c,d, Supporting Information), and compared with healthy controls, LINC00668 levels in the neutrophils of IBD patients were significantly increased and co‐localized in the nucleus with neutrophil elastase (NE) (Figure S3e,f, Supporting Information). These results indicate that the changes in LINC00668 expression and NETs content in IBD patients are similar to those seen in IBD mouse model, implying that LINC00668 exerts the same effect in IBD patients as in IBD mouse model. In future studies, we will provide further evidence indicating the clinical significance of exosomal‐LINC00668 in the NETs formation and IBD‐associated thrombosis.

### 2.6 LINC00668 Promotes the Translocation of NE from the Cytoplasmic Granules to the Nucleus and Induces NETs Release

**Figure 6:**
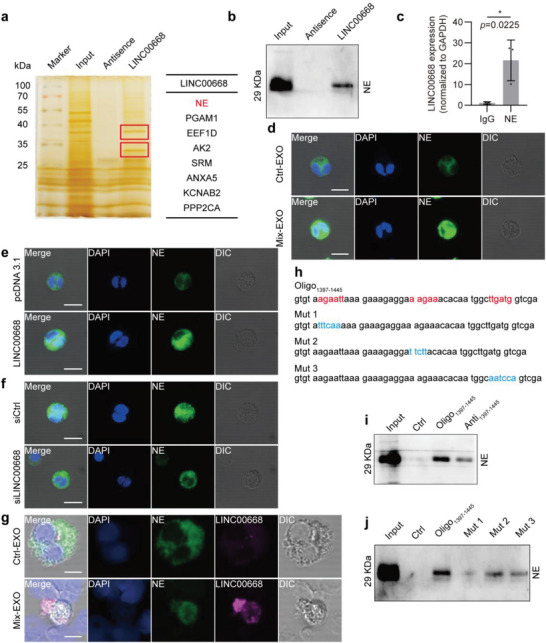
LINC00668 promotes the translocation of NE from the cytoplasmic granules to the nucleus and induces NETs release. a) The proteins interacting with LINC00668 in HL‐60 cells identified by biotinylated LINC00668 pull‐down followed by mass spectrometry. b) The proteins associated with LINC00668 in HL‐60 cells were pulled down by biotinylated LINC00668 or its antisence sequence (Antisence), and the precipitates were analyzed by Western blotting using anti‐NE antibody. c) qRT‐PCR detection of LINC00668 in the RNA‐protein immunoprecipitates pulled down by IgG or anti‐NE antibody in LINC00668‐overexpressing HL‐60 cells. d) Neutrophils were incubated with the exosomes isolated from Ctrl (Ctrl‐EXO) or Mix‐treated (Mix‐EXO) Caco‐2 cells, and immunofluorescence staining detected the subcellular localization of NE. Green and blue staining indicates NE and the nuclei, respectively. Differential Interference Contrast (DIC) shows 3D stereoscopic projection of cells. Scale bars represent 50 µm. e) Neutrophils were transfected with pcDNA3.1 or LINC00668‐overexpressing plasmids, and immunofluorescence staining detected the subcellular localization of NE. Green and blue staining indicates NE and the nuclei, respectively. Scale bars represent 50 µm. f) Caco‐2 cells were transfected with siCtrl or siLINC00668 and stimulated with Mix for 24 h. Exosomes were isolated from the culture supernatant and used to treat neutrophils, and NE subcellular localization was detected using immunofluorescence staining. Scale bar represents 50 µm. g) Immunofluorescence staining of NE and LINC00668 fluorescence in situ hybridization (FISH) on neutrophils treated with the exosomes isolated from Ctrl (Ctrl‐EXO) or Mix‐treated (Mix‐EXO) Caco‐2 cells. Green, magenta, and blue staining indicate NE, LINC00668, and the nuclei, respectively. Scale bars represent 50 µm. h) The sequence of 1397–1445 nucleotide positions in the LINC00668 sequence (Oligo_1397‐1445_) and the 3 mutants (Mut 1, Mut 2, and Mut 3) for the binding sites 1, 2, and 3 in Oligo_1397‐1445_ sequence. i) The lysates of HL‐60 cells were incubated with biotinylated Oligo_1397‐1445_, the antisense sequence of Oligo_1397‐1445_ (Anti_1397‐1445_) or Ctrl, and Western blot analysis detected NE level in the Oligo‐protein immunoprecipitates. j) HL‐60 cell lysates were incubated with biotinylated Oligo_1397‐1445_ or the mutants for the binding sites 1, 2, and 3 (Mut 1, Mut 2, and Mut 3), and Western blot analysis detected NE level in the Oligo‐protein immunoprecipitates. Data are represented as mean ± SEM. *p<0.05, *p*‐value was determined by unpaired two‐tailed Student's t‐test.

The above findings led us to investigate the underlying mechanism by which LINC00668 induces NETs formation. Considering that one of the known mechanisms by which lncRNAs exert their biological function is binding to specific protein partners,^[^ ^32^ ^]^ we used biotinylated full‐length LINC00668 to pull down the proteins that interact with LINC00668 from the lysates of HL‐60 cells, a human promyelocytic cell line. The mass spectrometry analyses of LINC00668‐associated proteins revealed that the 265 proteins were pulled down by the biotinylated LINC00668 (**Figure** **6**a). Among these proteins, NE, an azurin granule protein, has attracted our attention because of the translocation of NE from the cytoplasmic granules to the nucleus during NETs formation, where this enzyme cooperates with MPO to promote chromatin decondensation by cleaving histones.^[^ ^18^ ^]^ Further, RNA pull‐down followed by Western blot analysis revealed that NE could be pulled down by biotinylated LINC00668 (Figure 6b). Concurrently, RNA‐binding protein immunoprecipitation (RIP) assays demonstrated that LINC00668 was enriched in the complexes precipitated with antibodies against NE compared to those with control IgG (Figure 6c). Next, we sought to determine whether NE undergoes a nuclear translocation during the formation of NETs induced by Mix‐treated Caco‐2 cells‐derived exosomes (Mix‐EXO). Immunofluorescence staining showed that NE had a diffuse cytoplasmic distribution in Ctrl‐EXO‐treated neutrophils, but it was translocated to the nucleus when neutrophils were incubated with Mix‐EXO (Figure 6d). Similarly, overexpression of LINC00668 in neutrophils could also induce the nuclear translocation of NE (Figure 6e). However, the promoting effect of Mix‐EXO derived from LINC00668‐silenced Caco‐2 cells on the nuclear translocation of NE was largely abrogated (Figure 6f). These findings indicate that LINC00668 contained in Mix‐EXO facilitates the nuclear translocation of NE in neutrophils.

Several lncRNAs are known to contain nuclear‐localization signals, such as pentameric sequence AGCCC^[^ ^33^ ^]^ and a short sequence from the Alu element,^[^ ^34^ ^]^ and these specific sequences strictly regulate the subcellular localization of lncRNAs. However, these studies mostly focused on a certain cell itself. So, we wondered whether LINC00668 derived from the donor cells (IECs) can undergo a similar nuclear aggregation in recipient cells (neutrophils) during cell‐to‐cell communication mediated by exosomes. Therefore, we performed fluorescence in situ hybridization (FISH) for LINC00668 probe and immunofluorescence staining for NE antibody to examine the subcellular localization of NE and LINC00668. Compared with Ctrl‐EXO‐treated neutrophils, treating neutrophils with Mix‐EXO substantially enhanced the nuclear accumulation of LINC00668 and NE and increased their co‐localization in the nucleus (Figure 6g). Next, we tried to characterize the molecular basis of LINC00668‐NE interaction in neutrophils. First, we used the HDOCK (http://hdock.phys.hust.edu.cn/) to predict the potential interaction sites between LINC00668 and NE. As a result, 7 potential binding sites were predicted in LINC00668 sequence, of which the 1397–1445 nucleotide positions of the LINC00668 sequence densely clustered the three predicted sites (Figure 6h). We, therefore, selected the region containing these three potential binding sites for experimental verification. We synthesized a biotinylated fragment of 49 nts (Oligo_1397‐1445_) containing these sites and then incubated Oligo_1397‐1445_ with the lysates of HL‐60 cells. The results of RNA pull‐down followed by immunoblot analysis showed that NE was effectively pulled down by Oligo_1397‐1445_ (Figure 6i). As expected, the antisense sequence of Oligo_1397‐1445_ (Anti_1397‐1445_) could significantly attenuate the ability of LINC00668 to enrich NE in HL‐60 cells (Figure 6i). Next, to more precisely localize the interaction sites of LINC00668 with NE, we designed the three mutants (Mut 1, Mut 2, and Mut 3) for the binding sites 1, 2, and 3 in Oligo_1397‐1445_ sequence and labeled them with biotin. RNA‐pull down followed by Western blot analysis showed that the NE binding ability of the three mutants was reduced to varying degrees, and Mut 1 was almost completely unable to interact with NE. (Figure 6j). These results indicate that the binding site 1 (sequence 1402 to 1407 nts in LINC00668 sequence, agaatt) is the functional interaction site of LINC00668 and NE in neutrophils.

### 2.7 Berberine Inhibits NETs Formation through Blocking the Binding of LINC00668 to NE

**Figure 7:**
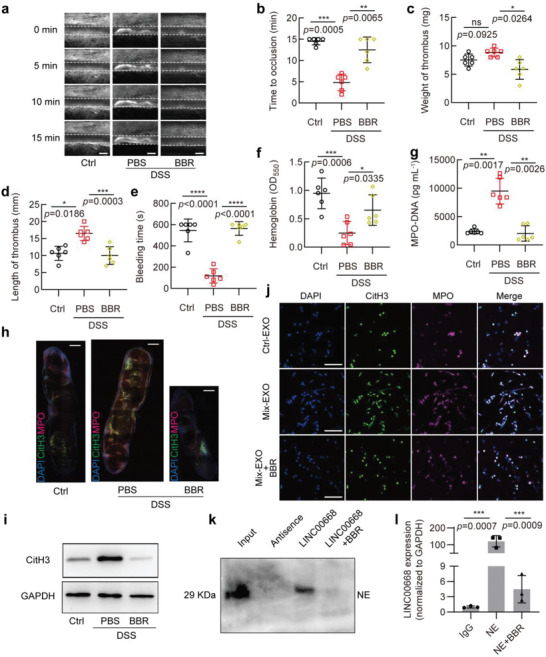
Berberine inhibits NETs formation by blocking the binding of LINC00668 to NE. a,b) Mesenteric arterioles ≈100 µm in diameter were injured with FeCl_3_ and white blood cells and platelets were labeled with Rhodamine 6G to detect thrombus formation by intravital microscopy. a) Representative images of thrombus formation in mice received drinking water alone (Ctrl) or treated with 3% DSS combined with PBS (PBS) or BBR (BBR) through intraperitoneal injection. Scale bars represent 100 µm. b) Vascular occlusion time in 3 groups of mice treated as in (a), with 6 mice in each group. c,d) Mice treated as in (a) were subjected to surgery for inferior vena cava stenosis, after 48 h, c) weight and d) length of thrombus harvested from 3 groups of mice (6 mice in each group) were measured. e) Tail bleeding time in mice treated as in (a), with 6 mice in each group. f) Blood loss was measured by the absorbance of hemoglobin at a wavelength of 550 nm using a microplate reader (6 mice in each group). g) MPO‐DNA ELISA was used to assess NETs in plasma of 3 groups of mice treated as in (a), with 6 mice in each group. h) CitH3 (green) and MPO (magenta) staining in the thrombus of inferior vena cava thrombosis in 3 groups of mice treated as in (a) (*n* = 3 mice per group). Scale bars, 500 µm. i) Western blot analysis of CitH3 expression in the whole thrombus obtained two days after surgery for inferior vena cava stenosis. j) Neutrophils were incubated with the exosomes isolated from Ctrl (Ctrl‐EXO) or Mix‐treated (Mix‐EXO) Caco‐2 cell and then treated or not with BBR. Immunofluorescence staining for CitH3 (green), MPO (magenta), and DAPI (blue) was performed on neutrophils. Scale bars represent 50 µm. k) HL‐60 cell lysates were pulled down by biotinylated LINC00668 with or without BBR or its antisence sequence (Antisence), and the precipitates were analyzed by Western blotting using anti‐NE antibody. l) qRT‐PCR detection of LINC00668 in the RNA‐protein immunoprecipitates pulled down by IgG or anti‐NE antibody in LINC00668‐overexpressing HL‐60 cells treated with or without BBR for 12 h. Results are presented relative to IgG immunoprecipitates. Data are represented as mean ± SEM, ns means no significant difference (*p* > 0.05), *p<0.05, **p<0.01, ***p<0.001, *p*‐value was determined by unpaired two‐tailed Student's t‐test.

To explore whether existing anti‐inflammatory and antithrombotic drugs inhibit LINC00668‐mediated nuclear translocation of NE and consequent NETs formation, we tested several drugs and found that BBR, which has been demonstrated to have the dual activities of the antithrombotic and anti‐inflammatory effects,^[^ ^35^ ^]^ significantly prolonged the time of the superior mesenteric artery occlusion induced by DSS treatment compared with those receiving PBS (**Figure** **7**a,b). Correspondingly, treatment of DSS‐induced mice with BBR significantly reduced the weight and length of the venous thrombus caused by inferior vena cava stenosis relative to those treated with PBS (Figure 7c, d). Moreover, the bleeding time measured by tail docking experiments was prolonged (Figure 7e), and the hemoglobin level was also elevated in BBR‐treated mice compared with PBS‐treated controls (Figure 7f).

Next, we tried to determine whether NET is a potential target for BBR to exert its antithrombotic effect. First, we detected the content of NETs in the plasma and venous thrombus of mice subjected to different treatments. The results showed that BBR treatment markedly suppressed DSS‐induced formation of the MPO‐DNA complexes in mouse plasma (Figure 7g), and co‐immunofluorescence staining for CitH3 and MPO revealed that the content of NETs in the thrombus was greatly decreased when DSS‐induced mice were treated with BBR (Figure 7h). Also, the levels of CitH3 in the whole thrombus were reduced following BBR treatment, as shown by Western blot analysis (Figure 7i). These results suggest that BBR can effectively suppress NETs release and consequent thrombus formation.

In the further experiments, neutrophils were incubated with the exosomes isolated from Ctrl (Ctrl‐EXO) or Mix‐treated (Mix‐EXO) Caco‐2 cells and then treated or not with BBR, and immunofluorescence staining for CitH3 and MPO was used to detect NETs formation. When neutrophils pretreated with BBR were incubated with Mix‐EXO, the neutrophils‐released NETs were largely diminished compared with those pretreated without BBR (Figure 7j). Subsequently, we investigated the effect of BBR on the interaction of LINC00668 with NE. The results showed that the recruitment of biotinylated LINC00668 to NE from the lysates of HL‐60 cells pretreated with BBR was significantly reduced, with the antisense sequence of LINC00668 being unable to pull down NE (Figure 7k). Consistently, the results of RIP followed by RT‐PCR indicated that the enrichment of NE to LINC00668 was attenuated in the BBR‐pretreated HL‐60 cells (Figure 7l). Taken together, these findings suggest that BBR suppresses the nuclear translocation of NE and subsequent NETs formation by inhibiting the interaction of LINC00668 with NE, indicating that NE inhibition is involved in the anti‐inflammatory and antithrombotic effect of BBR.

## 3 Discussion

Novel findings in the present study include the following: 1) NETs formation significantly increases in the DSS‐induced IBD mice; 2) Exosomes released by IECs under inflammation conditions contribute to thrombus formation through promoting neutrophils to release NETs; 3) LINC00668 highly enriched in the IECs‐derived exosomes mediates the translocation of NE from the cytoplasmic granules to the nucleus, thereby stimulating histone cleavage and chromatin decondensation, leading to NETs release; 4) BBR suppresses the nuclear translocation of NE and subsequent NETs formation via inhibiting the interaction of LINC00668 with NE, thus exerting its antithrombotic effects. These observations provide a mechanistic explanation for the linking IBD and thrombosis by exosome‐mediated NETs formation.

A large number of clinical studies have shown a strong association between IBD and thrombosis, yet very little basic research has been done on this phenomenon. Although UC is characterized by limited inflammation affecting the mucosal layer of the colon, and CD affects both the small intestine and the colon and tends to have more systemic inflammation, chronic inflammation of the intestinal tract is a common feature of both UC and CD. Thus, elucidation of the precise mechanisms of how chronic inflammation of the intestinal tract facilitates IBD‐associated thrombosis will provide a potential therapeutic target for the prevention and treatment of UC and CD‐associated thrombosis. Consistent with previous findings that DSS‐induced colitis increased thrombus formation in mouse arterioles in response to photochemical injury, which was significantly blunted in mice treated with the IL‐1β antibody as well as IL‐1r (‐/‐) mice,^[^ ^36^ ^]^ our data reproduced the clinical manifestation of IBD‐induced thrombosis in superior mesenteric artery and inferior vena cava, which was accompanied by severe inflammation. For the mediators that link the IBD‐associated thrombosis and the inflammatory response, Yan et al. showed that IL‐6‐infused mice exhibited an acceleration of thrombus formation in arterioles, similar to DSS.^[^ ^37^ ^]^ This was further confirmed by the observation of Senchenkova et al. that exogenous IL‐6 administered to mice elicited a dose‐dependent enhancement of thrombus formation.^[^ ^38^ ^]^ Another study showed that TNF‐α was implicated in the enhanced microvascular thrombosis that occurs during colonic inflammation, and the combined actions of TNF‐α and IL‐1β accounted for most of the colitis‐enhanced thrombotic response.^[^ ^39^ ^]^ Moreover, the DSS‐induced thrombosis was greatly attenuated in transgenic mice overexpressing the endothelial protein C receptor, and activated protein C afforded protection against thrombosis.^[^ ^40^ ^]^ These observations suggest that mechanism underlying the formation of thrombosis in IBD is very complex. With the discovery of NETs, the triggers of thrombosis were greatly enriched. Here, we confirmed that plasma levels of NETs were significantly elevated in mice with DSS‐induced colitis, which is in line with a recent study that NETs were induced in mice with DSS colitis and drived thrombotic tendency during active IBD.^[^ ^14^ ^]^ Notably, we demonstrated that the content of NETs in inferior vena cava thrombus of DSS‐induced colitis mice was significantly increased. After the degradation of NETs with DNase I, the length and weight of inferior vena cava thrombus were reduced, and the time of superior mesenteric artery occlusion was prolonged. These findings clearly suggest that elevated NETs, as a new mediator linking the inflammatory response of DSS colitis and thrombosis, may contribute to thrombosis.

The Induction of thrombosis by NETs was first discovered by Fuchs et al., who observed that NETs in vitro could aggregate and adsorb a large number of platelets, inducing red thrombus rich in red blood cells, and found the structure of NETs in iliac vein thrombosis in baboons.^[^ ^12a^ ^]^ Thereafter, large amounts of NETs were found in both human and animal arterial and venous thrombus. There has been a lot of evidence that NETs are associated with thrombosis. For example, NETs promote thrombin formation^[^ ^41^ ^]^ and provide a scaffold for thrombosis and growth.^[^ ^12a^ ^]^ Histones that makeup NETs are also conducive to thrombosis.^[^ ^42^ ^]^ In addition, NETs can also trigger internal and external coagulation pathways to activate platelets, thereby promoting thrombosis.^[^ ^13b^ ^]^ Here, we attempt to uncover the mechanism by which IBD causes an increased formation of NETs in plasma. Considering that IECs are not in direct contact with neutrophils, we hypothesized that exosomes released by IECs, as the mediator of long‐distance signaling for cell‐cell communication, might exert its effect on NETs formation. Previously, most studies on exosomes‐mediated thrombosis have explored extracellular vesicles secreted by hemocytes and endothelial cells that are in direct contact with blood. Among them, platelet‐derived extracellular vesicles have attracted the most attention because their surface‐externalized phosphatidylserine (PS) promotes the assembly of coagulation cascade components, contributing to thrombus formation together with the procoagulant protein tissue factor (TF).^[^ ^43^ ^]^ Unlike the link between IBD and blood clots, the link between cancer and blood clots was established as early as the 19^th^ century and has been more thoroughly studied, with many researchers finding TF exposure on the surface of extracellular vesicles secreted by cancer cells.^[^ ^44^ ^]^ Our study focused on the role of exosomes in bridging the gap between IBD and thrombosis. We confirmed that neutrophils produced NETs after being co‐cultured with Mix‐treated Caco‐2 cells. Remarkably, the exosomes isolated and purified from Caco‐2 cells treated with Mix also successfully induced neutrophils to release NETs. These suggest that inflamed IECs‐derived exosomes are responsible for NETs formation in IBD. Undoubtedly, the discovery of exosomes as pathogenic factors may have a positive effect on the treatment of IBD. For a long time, proinflammatory factors such as TNF‐α and IL‐6 in IBD patients have aroused people's attention, and monoclonal antibodies or small‐molecule drugs targeting specific cytokines or their receptors have been used to treat IBD. However, despite these therapeutic advances, clinical and endoscopic remission rates have reached a ceiling of 30% to 40%.^[^ ^45^ ^]^ Thus, it seems that there are other pathways affecting the inflammatory process, and NETs deserve further exploration. In addition to cytokines, exosomes contain other pro‐inflammatory factors such as lncRNAs, circRNAs, and miRNAs. Given the important role of lncRNAs in the regulation of various biological functions and pathological processes, we analyzed lncRNAs in IECs‐derived exosomes by using RNA‐seq and found that LINC00668 was highly enriched in the exosomes secreted by Mix‐treated Caco‐2 cells, but this lncRNA was not expressed in neutrophils. Using loss‐ and gain‐of‐function approaches, we demonstrated that exosomes mediated the transfer of LINC00668 from inflamed IECs into neutrophils and that an increased level of LINC00668 in recipient neutrophils was essential for NETs formation in the context of IBD. This study enriches the understanding of the molecular mechanism of exosome‐mediated thrombosis.

LINC00668 is upregulated in multiple cancer tissues and promotes tumorigenesis and metastasis by sponging miRNAs^[^ ^46^ ^]^ as well as by interacting with proteins.^[^ ^47^ ^]^ In this study, LINC00668 was significantly upregulated in exosomes secreted from Mix‐treated Caco‐2 cells and was delivered into neutrophils via IECs‐secreted exosomes, where it interacted with NE and facilitated the translocation of NE from the cytoplasm to the nucleus. NE, a serine protease stored in the azurophil granules of neutrophils, is well known to be able to cleave a variety of extracellular matrix proteins and degrade soluble proteins and proinflammatory mediators.^[^ ^48^ ^]^ Also, NE participates in intracellular pathogen destruction with potent antimicrobial activity.^[^ ^8^ ^]^ Moreover, NE is essential to initiate NET formation and it synergizes with MPO to drive chromatin decondensation.^[^ ^18^ ^]^ The classical explanation for NETs formation is that NETs are induced by NADPH oxidase‐dependent ROS, which triggers the release of NE and MPO from azurophilic granules,^[^ ^49^ ^]^ both of which translocate to the nucleus and cleave histones, causing chromatin decondensation.^[^ ^50^ ^]^ Another key factor, PAD4, enables arginine citrullination of histone residues, which reduces the positive charge of histones and their electrostatic interactions with DNA, allowing further relaxation of chromatin.^[^ ^50^ ^]^ NE and PAD4 may act in a synergistic or completely independent manner.^[^ ^51^ ^]^ The decomposing chromatin, accompanied by disassembly of the nuclear envelope, enters the cytoplasm and assembles with granular proteins to form NETs. Eventually, the NETs break through the plasma membrane and are extruded.^[^ ^52^ ^]^

Regarding the mode of translocation of NE from the granules to the nucleus, Metzler et al. showed that ROS triggers the dissociation of NE from a membrane‐associated complex into the cytosol and activates its proteolytic activity in an MPO‐dependent manner. In the cytosol, NE first binds and degrades F‐actin to arrest actin dynamics and then translocates to the nucleus.^[^ ^53^ ^]^ Despite these advances, the mechanism of cytosol‐to‐nucleus translocation of NE remains unclear. Here, we showed that the NE translocated to the nucleus from the granules by binding with LINC00668, as confirmed by RNA pull‐down and RIP experiments as well as by increased nuclear localization of LINC00668. These findings indicate that NE might be carried by LINC00668 from the cytoplasm to the nucleus. Indeed, we found that sequence 1402 to 1407 nts (agaatt) in LINC00668 sequence is responsible for the binding of LINC00668 to NE and the subsequent nuclear translocation of NE. Our finding provides an experimental basis for explaining the way NE enters the nucleus and provides a target for drug design. Previous studies have demonstrated that several lncRNAs, which are enriched in the cytoplasm or nucleus, bind to and regulate protein's subcellular localization, and that the subcellular localization of lncRNAs is very finely regulated as well. For example, Nguyen et al. found that a short interspersed nuclear element (SINE) in lncRNA Malat1 promotes Malat1 nuclear retention by facilitating its binding to HNRNPK. The loss of this RNA‐protein interaction caused by the SINE deletion may create more available TDP‐43 binding sites on Malat1 and subsequent TDP‐43 mislocalization, contributing to cancer and neurodegenerative diseases.^[^ ^54^ ^]^ The enrichment of lncRNAs in the nucleus depends on the nuclear localization sequences,^[^ ^55^ ^]^ such as the pentamer sequence “AGCCC”^[^ ^33^ ^]^ and the ALU‐rich sequence.^[^ ^34^ ^]^ Based on the findings that LINC00668 I into neutrophils from the exosomes could carry NE from the cytoplasm to the nucleus, we reasoned that LINC00668 may also contain a nuclear localization signal in its sequence. This would be a future experimental research direction.

At present, the therapeutic effect of IBD is not ideal. Although anti‐TNF‐α monoclonal antibody has become a therapeutic means to relieve IBD, ≈40% of patients have no response to anti‐TNF‐α therapy.^[^ ^56^ ^]^ In addition, the increased risk of severe infection^[^ ^57^ ^]^ and malignancy^[^ ^58^ ^]^ is a cause for concern. Targeting integrin and interleukin can serve as novel molecular treatment strategies to limit IBD progression, but they are expensive. Thus, it is urgent to find an effective and inexpensive drug that can effectively relieve the symptoms of IBD while reducing thrombosis. BBR is an isoquinoline alkaloid isolated from traditional Chinese medicines such as Huang Lian, Huang Bo, and Scutellaria, and has been used for many years to treat diarrhea, it is safe, effective, and inexpensive. Besides, BBR is also a clinically effective drug for the treatment of hyperlipidemia and type 2 diabetes.^[^ ^59^ ^]^ Recent study reported that BBR mitigates atherosclerosis by reducing Trimethylamine‐N‐oxide (TMAO).^[^ ^60^ ^]^ Moreover, BBR and its major metabolites have been shown to exert anti‐thrombotic actions through direct inhibition of thrombin,^[^ ^35c^ ^]^ or by reducing platelet activation.^[^ ^35b^ ^]^ The anti‐thrombotic function of BBR was further confirmed in this study, as arterial and venous thrombosis was significantly reduced in DSS‐induced colitis mice treated with BBR. We also explored the possible mechanisms of such an effect of BBR at the cellular level, where the interaction of LINC00668 with NE was blocked in BBR‐treated HL‐60 cells, and the release of NETs was significantly reduced. Also, the NETs content in thrombus in vivo was similarly decreased. These findings suggest that BBR inhibits the production of NETs by blocking the binding of LINC00668 to NE, providing new insights into the protective mechanism of BBR against IBD.

A major limitation of this study is the smaller number of IBD patients that we have detected in the present study. Another limitation is that, although we have demonstrated that BBR can effectively suppress NETs release and consequent thrombus formation through blocking the binding of LINC00668 to NE, the present study has not been able to identify that BBR is effective for the prevention and treatment of NETs formation and IBD‐associated thrombosis in IBD patients. These issues will be addressed in future research.

## 4 Experimental Section

### Animal Experiments

All animal studies were approved by the Institutional Animal Care and Use Committee and all efforts were made to minimize suffering. Epidemiological studies had shown that female IBD patients were more prone to thrombosis than males. In addition, female mice were better managed, tolerate the treatment, and had good experimental consistency and reproducibility, compared to male mice, so female BALB/c mice (Vital River Laboratories) aged 8 to 10 weeks were used for the study. To induce colitis, 3% Dextran sulfate sodium salt (Meilunbio, MB5535) dissolved in water was given to mice ad libitum for 7 days. DNase I (Solarbio, D8071) and berberine (Solarbio, B8500) were dissolved using sterile saline. For the DNase I treatment, mice were treated with DNase I (0.1U) every 3 days by tail vein injection. While, for the berberine treatment, mice were injected intraperitoneally with berberine (1 mg kg^−1^) every day. GW4869 (2.5 mg kg^−1^ day^−1^) (MCE, HY‐19363) dissolved in DMSO (Sigma, 01934) was diluted in sterile saline and treated mice by intraperitoneal injection, while controls were treated with equal amounts of DMSO. The Disease Activity Index (DAI) was a composite score of 3 aspects: weight loss (0%, 0–5%, 5–10%, 10–15%, >15%), stool characteristics (formed pellets, paste, dilute watery stool) and degree of blood in stool (no blood, positive occult blood, blood in the naked eye), which was used to represent the degree of disease in colitis.

### In vivo Microscopic Monitoring of FeCl_3_‐Induced Thrombosis in the Superior Mesenteric Artery

Observation of FeCl_3_‐induced thrombus formation in small mesenteric arteries of mice by in vivo microscopy as described with some modifications. After mice were anesthetized with 1.5% isoflurane (RWD), Rhodamine 6G (Sigma, 83697), and were injected through the tail vein into the blood of mice for 10 min to label platelets and leukocytes. 1 × 1 mm filter paper soaked in 5% FeCl_3_ (MACKLIN, I809489) was used to treat the superior mesenteric artery with a diameter of ≈100 µm. After 5 min of injury, the filter paper was removed and the residual FeCl_3_ was washed away with PBS. Vascular occlusion was monitored and retimed under a Leica inverted microscope (CTR7000 HS) for 15 min, and the body temperature of the mice was maintained during the experiment. Only one small artery was selected for each mouse.

### Inferior Vena Cava (IVC) Stenosis

Inferior vena cava stenosis was used to induce deep vein thrombosis. After the mice were anesthetized with isoflurane, the skin and peritoneum were incised along the midline of the abdomen to expose the abdominal cavity, and the intestine was removed and wrapped in gauze soaked in saline at 37 °C. All branches of the IVC were ligated, and the IVC was carefully separated from the aorta below the angle between the left renal vein and the IVC. The inferior vena cava was then ligated with a 7‐0 polypropylene suture along with a 30‐gauge needle, which was then removed and the IVC formed ≈90% stenosis, thus avoiding endothelial detachment. Sequential suturing of the peritoneum and skin. The thrombus was excised after 48 h for length and weight measurements.

### Tail Bleeding Assay

Mice anesthetized with isoflurane were placed on a heating plate to maintain body temperature, severed 5 mm from the tip of the tail to cause a coherent injury, and the tail was immediately immersed in 15 mL of 37 °C saline, and the bleeding time was recorded until bleeding stopped or until 10 min. Stopping bleeding for >1 min was considered as the end of bleeding, and the tail was removed promptly. The hemoglobin content was measured using a spectrophotometer to indicate the amount of blood loss in the mice. After removing the supernatant, 900 µL of water was added to resuspend and lyse the erythrocytes. After 17 s, 100 µL of 10 × PBS was added to restore the osmotic pressure and the cells and debris were removed by centrifugation (10 000 rpm, 5 min), and the supernatant containing hemoglobin was retained for optical density detection at 550 nm (OD550).

### MPO‐DNA ELISA

To quantify NETs in plasma, an MPO‐DNA enzyme‐linked immunosorbent assay (ELISA) was used as described previously. 5 µg mL^−1^ anti‐human MPO antibody (Proteintech, 22225‐1‐AP) was coated onto a 96‐well plate for capturing the MPO‐DNA complex, the DNA in the complex was detected with a peroxidase‐coupled anti‐DNA monoclonal antibody (Cell Death ELISAPLUS, Roche; dilution 1:25), and the absorbance at 405 nm was measured after 20 min incubation with peroxidase substrate.

### Histological Analysis

After euthanasia, the terminal colon at 1–2 cm from the anus was removed, fixed in 4% paraformaldehyde for 24 h, dehydrated in gradients of different concentrations of alcohol and xylene solutions, and dipped in paraffin for 2 h to make paraffin sections. Cross‐sectional colon sections of 4 µm thickness were used for histological analysis, then, hematoxylin and eosin staining were performed sequentially after gradient alcohol hydration with xylene dewaxing. Images were acquired using a Leica microscope (Leica DM6000B, Germany) and digitized with LAS V.4.4 (Leica). Histological scoring was performed on colon sections, and the score consisted of the sum of five subscales: including mononuclear cell infiltration (0‐3), crypt hyperplasia (0‐3), epithelial damage (0‐3), polymorphonuclear cell infiltration (0‐3), and transmural inflammation (0‐4).

### Immunohistochemistry and Immunofluorescence

Longitudinal sections of thrombus sections were used for analysis. After dewaxing and hydration, the antigen was repaired in citrate solution under high pressure for 3 min. For immunohistochemistry, endogenous peroxidase was inactivated with H_2_O_2_, 5% goat serum was closed at room temperature for 30 min, and primary antibody rat anti‐Ly‐6G (Abcam, ab25377) was used in combination with HRP goat anti‐rat IgG polymer detection kit (ZSGB‐BIO, PV‐9004), and finally hematoxylin re‐staining was performed. For immunofluorescence, For immunofluorescence, incubated with 1% BSA (Thermo Scientific, 37520) for 30 min at room temperature to block non‐specific binding, primary antibodies anti‐histone H3 (Abcam, ab5103), human/mouse myeloperoxidase/MPO antibody (R&D Systems, AF3667‐SP) and anti‐neutralizing elastase (Abcam, ab131260) incubated overnight at 4 °C, secondary antibodies fluorescein (FITC)‐conjugated Affinipure donkey anti‐rabbit IgG (H+L) (Proteintech, SA00003‐8), rhodamine (TRITC)‐conjugated donkey anti‐goat IgG (H+L) (Proteintech, SA00007‐3) and fluorescein (FITC)‐conjugated Affinipure sheep anti‐goat IgG (H+L) (Proteintech, SA00003‐2) were incubated for 1 h at 37 °C and 4′,6‐diamidino‐2′‐phenylindole dihydrochloride (DAPI)(S outhernBiotech, 0100–20) was used to stain the nucleus. Images were acquired using a Leica microscope (Leica DM6000B, Germany).

### Isolation of Human Neutrophils

Human peripheral blood samples were collected from healthy donors, and the study protocol was approved by the ethics review committee, with the informed consent of all participants. Neutrophils were isolated using the Human Peripheral Blood Neutrophil Isolation Kit (Solarbio, P9040) according to the manufacturer's instructions. Briefly, EDTA‐anticoagulated blood from healthy human donors was spread on a gradient separator and centrifuged (1000 g, 30 min) at room temperature. The neutrophil layer between the separators was carefully aspirated and washed with PBS, then equilibrated in pre‐warmed IMDM complete medium for 30 min before use in experiments.

### Fluorescence In Situ Hybridization

FISH assays were performed in neutrophils according to the manufacturer's instructions. The Cy3‐labeled LINC00668 probe used in this study was designed and synthesized by GenePharma (Shanghai, China). Briefly, neutrophils were fixed with 4% paraformaldehyde for 15 min. After permeabilization with 0.1% TritonX‐100 for 15 min, the cells were incubated with the specific probe overnight at 37 °C. The probe was washed off, and primary antibody Anti‐Neutrophil Elastase (Abcam, ab131260) was added and incubated overnight at 4 °C. Secondary antibodies were used Fluorescein (FITC)‐conjugated Affinipure Goat Anti‐Rabbit IgG(H+L) (Proteintech, SA00003‐2) at 37 °C for 1 h. Nucleus were stained with DAPI. Images were acquired using a Leica microscope (Leica Dmi8, Germany) and digitized with the software of LAS X (Leica).

### Cell Culture and Isolation of Extracellular Vesicles from Plasma or Cell Culture

Colorectal adenocarcinoma cells (Caco‐2 cells) were cultured in MEM medium (Gibco, C11095500BT) and human promyelocytic leukemia cells (HL‐60 cells) were cultured in IMDE medium (Gibco, C12440500BT). The above medium contains 100 U mL^−1^ penicillin, 100 µg mL^−1^ streptomycin and 10% fetal bovine serum (BIOEXPLORER LifeSciences, BS1615‐115). All cells were cultured in a humidified incubator at 37 °C with 5% CO_2_. Caco‐2 cells and HL‐60 cells were purchased from the cell bank of the Chinese Academy of Sciences (Shanghai, China). To avoid the interference of exosomes in FBS, Caco‐2 cells were replaced with MEM medium containing 2% Exosome‐depleted FBS (SBI, EXO‐FBS‐50A‐1) at 70–80% growth fusion for 24 h. The medium was treated with LPS (10 µg mL^−1^) (Solarbio, L8880), TNF‐α (50 ng mL^−1^) (Proteintech, HZ‐1014‐10), IL‐1β (25 ng mL^−1^) (Proteintech, HZ‐1164), IFN‐γ (50 ng mL^−1^) (Proteintech, HZ‐1301), respectively, as well as with a mixture of these four factors for 24 h to induce inflammation. The medium was washed twice with PBS to remove residual inflammatory factors and replaced with a fresh MEM medium containing 2% Exosome‐depleted FBS (SBI, EXO‐FBS‐50A‐1) for 24 h. The medium was collected. Dead cells and cell debris were first removed by centrifugation (2000 g, 15 min) at 4 °C, and the supernatant was collected and centrifuged at 20 000 g, 4 °C for 40 min to pellet the microvesicles. The supernatant was collected for exosome isolation. The pelleted microvesicles were resuspended by sterile ice‐cold PBS and pelleted by centrifugation again, the supernatant was discarded, and the final microvesicles were resuspended with 100 µL PBS. To obtain pure exosomes, the collected supernatant was filtered through a 0.22 µm membrane, and the filtered medium supernatant was concentrated to 3 mL and then spread flat onto a gradient of iodixanol (40%, 20%, 10%, 5% from bottom to top) and centrifuged using an ultracentrifuge (Hitachi, CP70ME) at 120 000 g at 4 °C for 18 h. The exosomes would be enriched in 20%−40% iodixanol. The fraction was aspirated with PBS to 8 mL and centrifuged again at 120 000 g for 70 min at 4 °C to pellet the exosomes. Finally, the purified exosome pellet was resuspended in 100 µL of sterile 1 × PBS. The above extracellular vesicles should be used as soon as possible and stored at 4 °C for no >3 days and at −80 °C for no more than one month.

### Electron Microscopy

For transmission electron microscopy (TEM), aspirated a drop of the sample suspension onto a copper mesh with a membrane, let it stand for 1 min, then aspirated the excess liquid with filter paper, stained with uranium peroxide acetate for 1–2 min, and aspirated the negative staining solution with filter paper, let it dry and used it for transmission electron microscopy (Hitachi, HT‐7700) to detect imaging.

### Nanoparticle Tracking Analysis (NTA)

The exosome particle size and concentration were measured using NTA at VivaCellBiosceinces with ZetaView PMX 110 (Particle Metrix, Meerbusch, Germany) and corresponding software ZetaView 8.04.02. The ZetaView system was calibrated using 110 nm polystyrene particles.

### Western Blotting

After cryogenic grinding and sonication of tissues or cells in RIPA lysate, the proteins were separate by SDS‐PAGE and transferred to PVDF membrane (Millipore), Membranes were blocked in TBS‐T containing 5% bovine serum albumin (BSA) at room temperature for 1 h, and incubated with primary antibodies overnight at 4 °C. Antibodies that were used are anti‐Histone H3 (1:1000 ab5103), anti‐p65 (1:1000, ab32536), anti‐IκB (1:1000, 10268‐1‐AP), anti‐Lamin B1 (1:1000, ab133741), anti‐GAPDH (1:10 000, 60004‐1‐Ig‐100), anti‐Alix (1:1000, ab186429), anti‐TSG101 (1:1000, ab125011), anti‐CD9 (1:1000, ab236630), anti‐Calnexin (1:1000, BS1438) and anti‐NE (1:1000, ab131260). After washing with TBST, the membrane was incubated with the HRP‐conjugated secondary antibody (1:10 000) for 1.5 h at room temperature. The blots were treated with the Immobilon Western (Millipore), and detected by ECL (enhanced chemiluminescence) Fuazon Fx (Vilber Lourmat). Images were captured and processed by FusionCapt Advance Fx5 software (Vilber Lourmat).

### ELISA

Quantification of inflammatory factors in the cell culture medium was determined by AuthentiKine Human IL‐6 ELISA Kit (Proteintech, KE00139) and AuthentiKine Human IL‐8 ELISA Kit (Proteintech, KE00275). The method was performed according to the manufacturer's instructions. Briefly, the cell supernatant was diluted 8‐fold and added to a 96‐well plate coated with primary antibody and incubated for 2 h at 37 °C. The enzyme‐labeled antibody HRP‐conjugated antibody was then added and incubated at 37 °C for 40 min. The reaction was incubated with the substrate solution for 3–5 min, measured the absorbance at 450 nm.

### RNA isolation and PCR

Total RNA was extracted from cells or exosomes using the E.Z.N.A. Total RNA Kit II (Omega, R6934‐02) according to the manufacturer's instructions. RNA concentrations were measured using a Nanodrop 2000 (Thermo). Reverse transcription of total RNA was performed using RevertAid First Strand cDNA Synthesis Kit (Thermo Scientific, K1622), qRT‐PCR of mRNA and lncRNA was done using MonAmp ChemoHS qPCR Mix (Monad, MQ00401S). The relative amounts of transcripts were normalized with GAPDH and calculated using the 2‐ΔΔCt formula. All samples were repeated three times.

### Measuring Generation and Quantitation of NETs

NET formation and quantitation were measured using the SYTOX Green (Invitrogen, S7020). Co‐culture of the two cell types was achieved by Transwell inserts (Corning), with Caco‐2 cells in the upper layer and neutrophils in the lower layer. The 0.4 µm membrane between the layers allowed exosomes to pass freely. Before co‐culture, Caco‐2 cells were treated with Mix for 24 h, the medium was discarded, and the cells were then washed twice with PBS, and supplemented with fresh medium, followed by co‐culture with the lower layer of neutrophils for 4 h. Paraformaldehyde at a final concentration of 4% was added to the medium, fixed for 20 min, permeabilized and stained with 1 µM SYTOX Green for 5 min, excess dye was washed away with PBS and put under the microscope for observation, and cloud‐like or thread‐like structures were extracellular DNA. Neutrophils were spread into 96‐well plates with both 30 nM SYTOX Green and exosomes (1 × 10^6^ particles per cell) and green fluorescence was detected at Ex485, Em520 after 4 h.

### RNA‐seq

High‐throughput sequencing was performed in Sinotech Genomics Co., Ltd. (Shanghai, China). To perform RNA‐seq analysis, total RNA was first extracted from exosomes of Caco‐2 cells, which was quality‐checked by NanoDrop ND‐2000 spectrophotometer and Agilent Bioanalyzer 4200 (Agilent Technologies, Santa Clara, CA, US), and the quality‐checked RNA was used for library construction. Then the libraries were sequenced on the Illumina HiSeq 2000 platform.

### Plasmid Constructs and Transfection

The overexpression plasmid, Oligo, and short interfering RNA (siRNA) of LINC00668 were designed and synthesized by GenePharma (Shanghai, China). Gene transfections were performed using Lipofectamine 2000 Transfection Reagent (Thermo Fisher, USA) according to the manufacturer's protocols.

### RNA pull‐down and Mass Spectrometric Analysis

To analyze the proteins interacting with LINC00668, the Pierce Magnetic RNA‐Protein Pull‐Down Kit (Thermo Fisher, 20164) was used for RNA pull‐down experiments, referring to the reagent manufacturer's instructions. Briefly, the Pierce RNA 3´ End Desthiobiotinylation Kit (Thermo Fisher, 20163) was used to label biotin on LINNC00668 to enable it to bind to the magnetic beads, and then the magnetic beads bound with full‐length LINC00668 were incubated with protein lysate from HL‐60 cells at 4 °C, and the proteins interacting with LINC00668 were pulled down. Finally, these proteins were analyzed using mass spectrometry.

### Crossed‐linked RNA Immunoprecipitation (RIP)

RIP experiments were performed using Dynabeads Protein G Immunoprecipitation Kit (Invitrogen, 10007D) according to the manufacturer's protocol. Briefly, HL‐60 cells were fixed in 1% formaldehyde for 10 min at room temperature and the cross‐linking reaction was stopped by adding 2.5 m glycine. Subsequently, NP‐40 lysate (Solarbio, N8032) was added and lysed on ice for 15 min, 30 µL of lysate was retained as input and equal amounts of protein lysate were incubated with magnetic beads coupled with anti‐IgG or anti‐NE antibodies (Abcam, ab131260). Immunoprecipitated RNA was extracted with E.Z.N.A. Total RNA Kit II (Omega, R6934‐02) and then reverse transcribed to cDNA and analyzed by qRT‐PCR.

### Statistical Analysis of Experimental Data

GraphPad Prism 9 software (GraphPad Software, San Diego, CA, USA) was used for statistical analysis. For continuous variables, the student t‐test or Mann‐Whitney U test was used for the comparison between the two groups. For the comparison of >2 groups, one‐way ANOVA with Dunnett's post hoc correction. A value of *p*<0.05 was considered statistically significant and denoted with 1, 2, 3, or 4 asterisks when <0.05, 0.01, 0.001, or 0.0001, respectively.

## Conflict of Interest

The authors declare no conflict of interest.

## Author Contributions

J.‐k.W. conceived the project, designed and supervised the research, and wrote the manuscript. L.Z. designed the experiment, performed the animal experiments and cell experiments, analyzed data, and interpreted the results. B.Z. designed and conducted experiments. Y.B. performed the animal model construction. Y.‐q.Y. carried out neutrophils and exosome isolation. J.Z. and J.Y. collected and analyzed the clinical data. X.‐h.Z. and H.‐y.Z. conducted cell culture and quantitative PCR. D.M. performed in vivo microscope observation. H.W. completed the collection of clinical samples.

## Supporting information

Supporting Information

## Data Availability

The data that support the findings of this study are available from the corresponding author upon reasonable request.
