# Supplementary material for: Exosomes‐transferred LINC00668 Contributes to Thrombosis by Promoting NETs Formation in Inflammatory Bowel Disease
Source: Adv Sci (Weinh). 2023 Aug 17;10(28):2300560. doi: 10.1002/advs.202300560 (PMC10558653; doi:10.1002/advs.202300560)
Supplement: Supplementary file 1 — Supporting Information [file ADVS-10-2300560-s001.pdf]

ADVANCED SCIENCE
Open Access

Supporting Information

for *Adv. Sci.*, DOI 10.1002/advs.202300560

Exosomes-transferred LINC00668 Contributes to Thrombosis by Promoting NETs Formation in Inflammatory Bowel Disease

Long Zhang, Bin Zheng, Yang Bai, Jing Zhou, Xin-hua Zhang, Yu-qin Yang, Jing Yu, Hong-ye Zhao, Dong Ma, Han Wu and Jin-kun Wen**

Exosomes-transferred LINC00668 Contributes to Thrombosis by Promoting NETs Formation in Inflammatory Bowel Disease

Long Zhang[1], Bin Zheng[1], Yang Bai[1], Jing Zhou[2], Xin-hua Zhang[1,4], Yu-qin Yang[1], Jing Yu[3], Hong-ye Zhao[1], Dong Ma[1], Han Wu[1], Jin-kun Wen[1]**

Table S1

	WBC [10^9 L^{-1}]	Lymphocyte [10^9 L^{-1}]	Monocyte [10^9 L^{-1}]	Neutrophil [10^9 L^{-1}]	RBC [10^12 L^{-1}]	Platelet [10^9 L^{-1}]
Ctrl 1	4.9	3.9	0.1	0.9	8.74	568
Ctrl 2	3.1	2.5	0.1	0.5	5.63	590
Ctrl 3	4.4	3.5	0.1	0.8	8.33	517
Ctrl 4	4.3	3.5	0.1	0.7	8.29	522
Ctrl 5	3.2	2.3	0.1	0.8	8.30	535
Ctrl6	3.9	2.9	0.1	0.9	9.02	495
DSS 1	50.1	41	1.7	7.4	8.57	705
DSS 2	39.1	31.9	1.3	5.9	4.74	419
DSS 3	51.2	42.2	1.8	7.2	7.94	540
DSS 4	54.1	45.1	1.8	7.2	8.41	886
DSS 5	49	39.5	1.5	8	7.91	460
DSS 6	46.6	38.6	1.6	6.4	7.72	649

Table S1. Blood cell analysis of mouse peripheral blood. Peripheral blood of mice was collected using EDTA anticoagulant collection vessel, and blood cells were detected immediately after being mixed upside down.

Figure S1

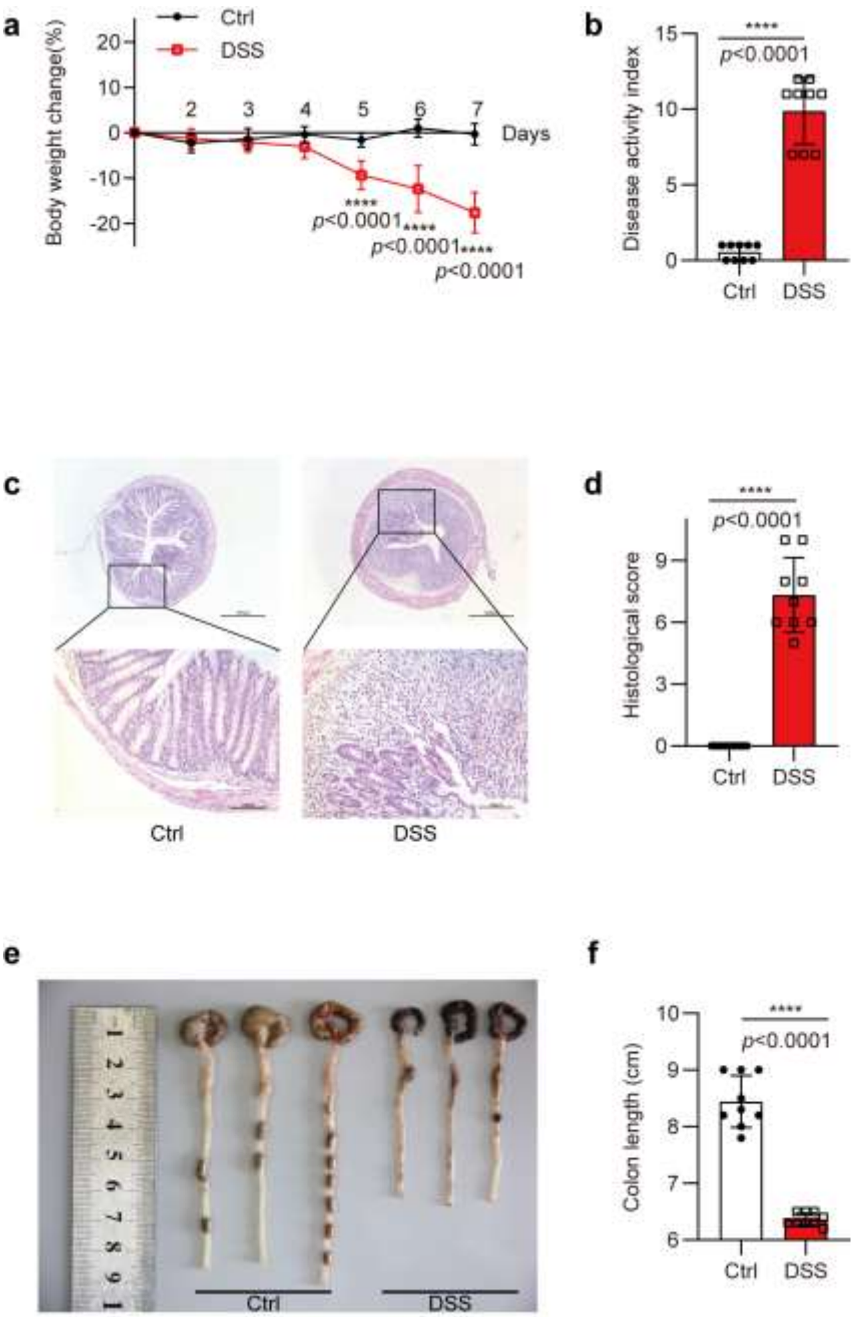

Figure S1. Establishment of mouse model of IBD. a) Changes in body weight of DSS-treated BALB/c mice over 7 days. Mice were given water alone (Ctrl), or water with 3% DSS (DSS) (9 mice/group) and body weight was assessed every day. Results were expressed as the percentage of weight loss relative to the initial weight. b) Disease activity index (DAI) in untreated mice and those treated with 3% DSS

measured at 7 d after treatment (9 mice/group). c) Representative HE staining image, and (d) histopathological score of distal colonic sections from Ctrl and 3% DSS-treated mice on day 7 (9 mice/group). e) Representative images of the colon and (f) the change of the colon length of Ctrl and DSS-treated mice (9 mice/group). Statistical analysis was performed using two-way ANOVA with Šídák's post hoc correction unpaired two-tailed Student's t-test, ****p<0.0001.

Figure S2

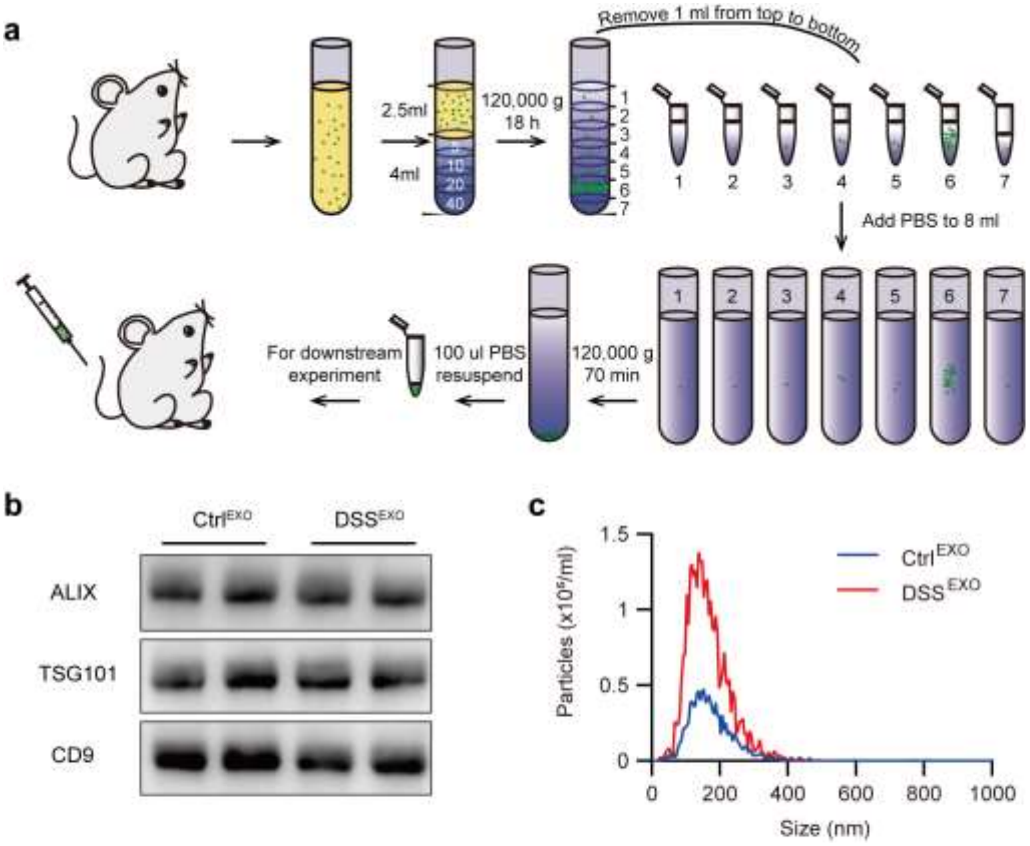

Figure S2. Isolation, identification and quantification of plasma exosomes. a) Anticoagulated blood collected from Ctrl and 3% DSS-treated mice was centrifuged at 3000 rpm for 15 min. 2.5 ml of supernatant plasma was loaded to an iodixanol gradient solution and centrifuged at 120,000 g for 18 h. Exosomes between 20% and 40% iodixanol were collected and washed once with PBS and then injected into the mice through the tail vein. b) Western blot analysis for the exosome markers ALIX, TSG101 and CD9. c) Quantification of plasma exosomes using NTA.

Figure S3

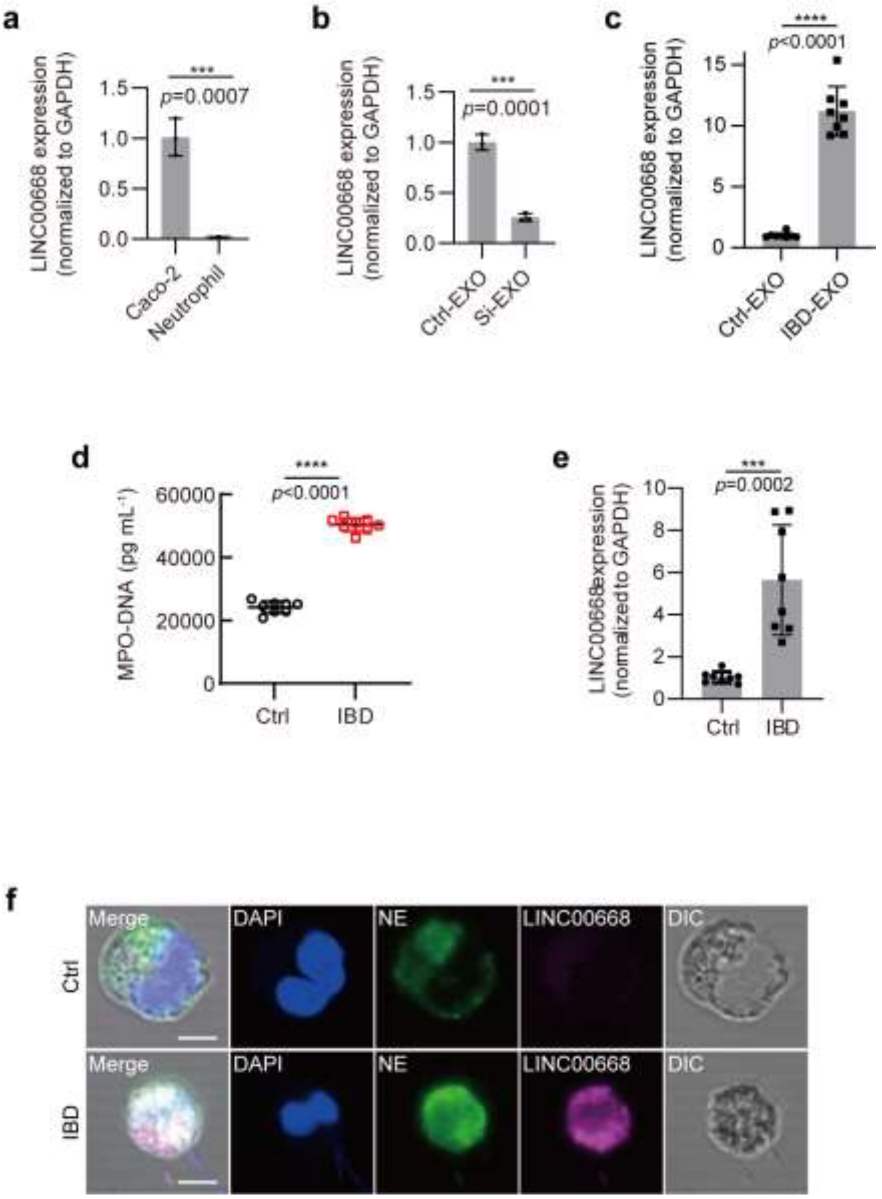

Figure S3. The knockdown and overexpression of LINC00668 in exosomes derived from Caco-2 cells. a) qRT-PCR detected LINC00668 expression in Caco-2 cells and neutrophils. b) LINC00668 was knocked down in Caco-2 cells, and the exosomes were collected from their culture supernatant, and the expression level of LINC00668 in exosomes was detected by using qRT-PCR. c) qRT-PCR detection of LINC00668 in plasma exosomes of IBD patients (n=8) and healthy controls (n=8). d) MPO-DNA ELISA was used to assess NETs in plasma of IBD patients (n=8) and healthy controls

(n=8). e) qRT-PCR detection of LINC00668 in neutrophils of IBD patients (n=8) and healthy controls (n=8). f) Immunofluorescence staining of NE and LINC00668 fluorescence in situ hybridization (FISH) on neutrophils isolated from whole blood of IBD patients and healthy controls. Green, red and blue staining indicates NE, LINC00668 and the nuclei, respectively. Scale bars represent 50 μm. Data are represented as mean ± SEM. ***$p<0.001$, p-value was determined by unpaired two-tailed Student's t-test.
